# Feasibility of ctDNA in detecting minimal residual disease and predicting recurrence for colorectal cancer liver metastases

**DOI:** 10.3389/fonc.2024.1418696

**Published:** 2024-10-08

**Authors:** Jennifer A. Kalil, Lucyna Krzywon, Stephanie K. Petrillo, Migmar Tsamchoe, Oran Zlotnik, Anthoula Lazaris, Peter Metrakos

**Affiliations:** ^1^ Department of Surgery, Royal Victoria Hospital - McGill University Health Center, Montréal, QC, Canada; ^2^ Research Institute, McGill University Health Center, Montréal, QC, Canada; ^3^ Department of Anatomy and Cell Biology, McGill University Health Center, Montréal, QC, Canada

**Keywords:** colorectal cancer liver metastases (CRLM), minimal residual disease (MRD), ctDNA, digital PCR, disease recurrence, liquid biopsy, liver resection

## Abstract

**Introduction:**

Approximately 50% of patients diagnosed with colorectal cancer develop colorectal cancer liver metastases (CRLM). Although curative intent liver resection provides 5-year survival of 40-50%, up to 70% of patients develop recurrence of CRLM. Detection of minimal residual disease (MRD) is essential for timely, optimized treatment. This study evaluated the feasibility and utility of using circulating tumor DNA (ctDNA) to identify MRD and predict disease recurrence.

**Methods:**

Patients with CRLM that underwent liver resection and had known KRAS or PIK3CA mutations were retrospectively identified. Serial blood samples were collected every 3 months following surgery for disease surveillance. ctDNA was isolated from the samples and analyzed with digital PCR (dPCR).

**Results:**

KRAS and PIK3CA mutations were identified by dPCR in 29 patients over 115 timepoints. In patients with detectable ctDNA at time of liver resection, 81% (13/16) developed disease recurrence, while 46% (6/13) of the patients with undetectable ctDNA recurred (p=0.064). Presence of ctDNA was detected in 27.6% (8/29) of the initial postoperative samples. Radiologic recurrence was later diagnosed in 100% (8/8) of these patients, while 52% (11/21) who had undetectable ctDNA postoperatively recurred (p=0.026). Detectable ctDNA postoperatively was associated with a shorter disease-free survival (DFS) of 9 months vs 13 months in patients who had undetectable ctDNA (HR 2.95, 95% CI 1.16-7.49; p=0.02).

**Conclusion:**

Liquid biopsy using dPCR can identify low levels of ctDNA, enabling early detection of disease recurrence. Additionally, the presence of ctDNA postoperatively was predictive of recurrence. This study corroborates current literature and provides rational for moving toward a clinical trial using ctDNA and dPCR to detect MRD after CRLM resection.

## 1 Introduction

Colorectal cancer is the third most common cause of cancer and cancer-related mortality worldwide (1). Approximately 50% of patients will develop colorectal cancer liver metastasis (CRLM) over the course of their disease and surgical resection remains the only treatment option capable of providing a cure or long-term survival (2, 3). Liver resection combined with perioperative systemic chemotherapy, either given in the neoadjuvant or adjuvant setting or a combination, has become the standard management for CRLM (4). Although curative intent liver resection provides 5-year survival of 40% - 50%, recurrence rates post-hepatectomy are 50% - 70% and mostly occur within the first two years with limited benefit from chemotherapy (5–8). Most clinical practice guidelines for post-treatment surveillance include frequent follow-up visits, cross sectional imaging of the chest, abdomen, and pelvis and carcinoembryonic antigen (CEA) testing for at least 5 years to monitor for recurrence (9–11). However, there is currently no validated biomarker that is being used routinely in post-treatment surveillance regimens to predict recurrence and guide adjuvant therapy. Minimally invasive approaches to identify patients with minimal residual disease (MRD) after treatment are being developed to identify patients with cancer who are at a risk of recurrence and monitor response to treatment. Predicting disease response and relapse has considerable potential for increasing the effective implementation of neoadjuvant and adjuvant therapies.

Many studies have demonstrated alterations in the cancers DNA are identified at a similar rate by tissue vs plasma genotyping (12). Liquid biopsy panels, detecting circulating tumor DNA (ctDNA), can play an important role in detection of minimal disease and overall prognosis because it can identify tumor mutations and predict therapeutic outcomes (12). Such an approach is unique from the current standard of obtaining a tissue biopsy as it is minimally invasive, can be utilized for repeated samplings at different time points during tumor progression and treatment. Circulating tumor DNA (ctDNA), detected with a peripheral blood sample, is released from both primary and metastatic sites of disease into the bloodstream during tumor growth and after apoptosis and has shown potential in serving as a prognostic marker (13, 14). Cancer-specific information, including point mutations, DNA methylation, and microsatellite instability (MSI), can be acquired through real-time or digital polymerase chain reaction (rtPCR, dPCR) and targeted sequencing techniques (15, 16). In the nonmetastatic setting, monitoring ctDNA post-operatively has shown promise in identifying minimal residual disease, predicting recurrence, survival, and guiding treatment although a similar role as a prognostic biomarker in CRLM remains uncertain with limited studies (15, 17). This is important as early detection of relapse using ctDNA could indicate patient populations where earlier therapeutic intervention may be beneficial.

By corroborating previously reported findings, this study contributes to the credibility of ctDNA as a prognostic biomarker in patients with CRLM. This study aims to validate the clinical utility of ctDNA analysis using digital PCR (dPCR) in predicting overall survival (OS) and disease-free survival (DFS) in selected patients with known KRAS and/or PIK3CA mutations who have undergone curative-intent liver resection for CRLM.

## 2 Methods

### 2.1 Patient selection and study design

**Figure 1:**
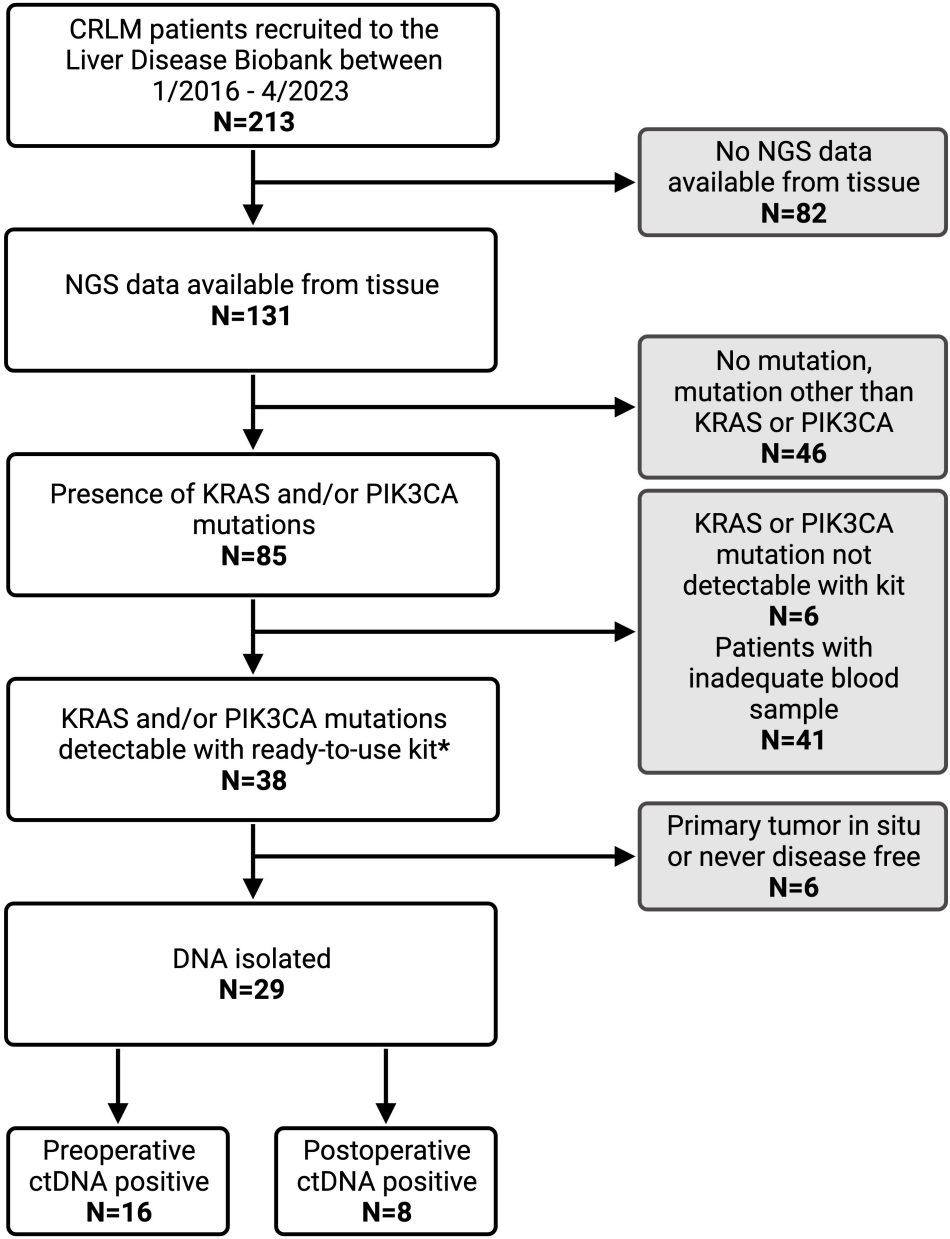
Patient inclusion criteria flow chart with reasons for exclusion from analysis. CRLM, colorectal liver metastasis; NGS, next generation sequencing; ctDNA, circulating tumor DNA. * IDKRAS ready-to-use kit detects KRAS mutation in codon G12, G13, and Q61. IDPIK3CA ready-to-use kit detects PIK3CA mutations at E542K, E545K, Q546K, H1047R and H1047L.

This single-center study retrospectively evaluated patients who had consented to participate in the Liver Disease BioBank at the McGill University Health Center. The study was conducted in accordance with McGill University Health Centre (MUHC) Research Ethics Board (REB) guidelines. Prior written informed consent was obtained from all subjects to participate in the study (protocol: SDR-11-066). Patients that underwent curative-intent liver resection for CRLM between 2016 and 2023 were identified. Only patients with next generation sequencing (NGS) data available from tissue samples were included. Furthermore, only those with KRAS and/or PIK3CA mutations were eligible. Patients whose primary tumor had been resected or was deemed resectable as a combined approach with liver resection were included. Patients with extrahepatic disease other than pulmonary nodules were excluded from the study (**Figure 1**).

Demographic and clinicopathologic data were retrospectively collected. Liver metastasis diagnosis and disease recurrence were determined radiologically. Overall survival was measured from the date of liver metastasis diagnosis to the date of death or last clinic visit, at which point the data were censored. Disease free survival was measured from the date of liver resection to the date of disease recurrence.

All patients received treatment and surveillance in accordance with the National Comprehensive Cancer Network (NCCN) Guidelines including follow up every 3-6 months with thoracoabdominal cross sectional imaging and serial measurement of CEA (2).

### 2.2 Tissue next generation sequencing

All NGS data was obtained from the patient medical record and is performed routinely as per clinical practice. NGS was performed on formalin-fixed paraffin-embedded (FFPE) tissue from either the primary tumor or metastasis using the AmpliSeq™ Focus Panel for Illumina^®^, which is a targeted sequencing assay for analysis of 52 genes with significance in solid tumors.

### 2.3 Sample collection and plasma isolation

**Figure 2:**
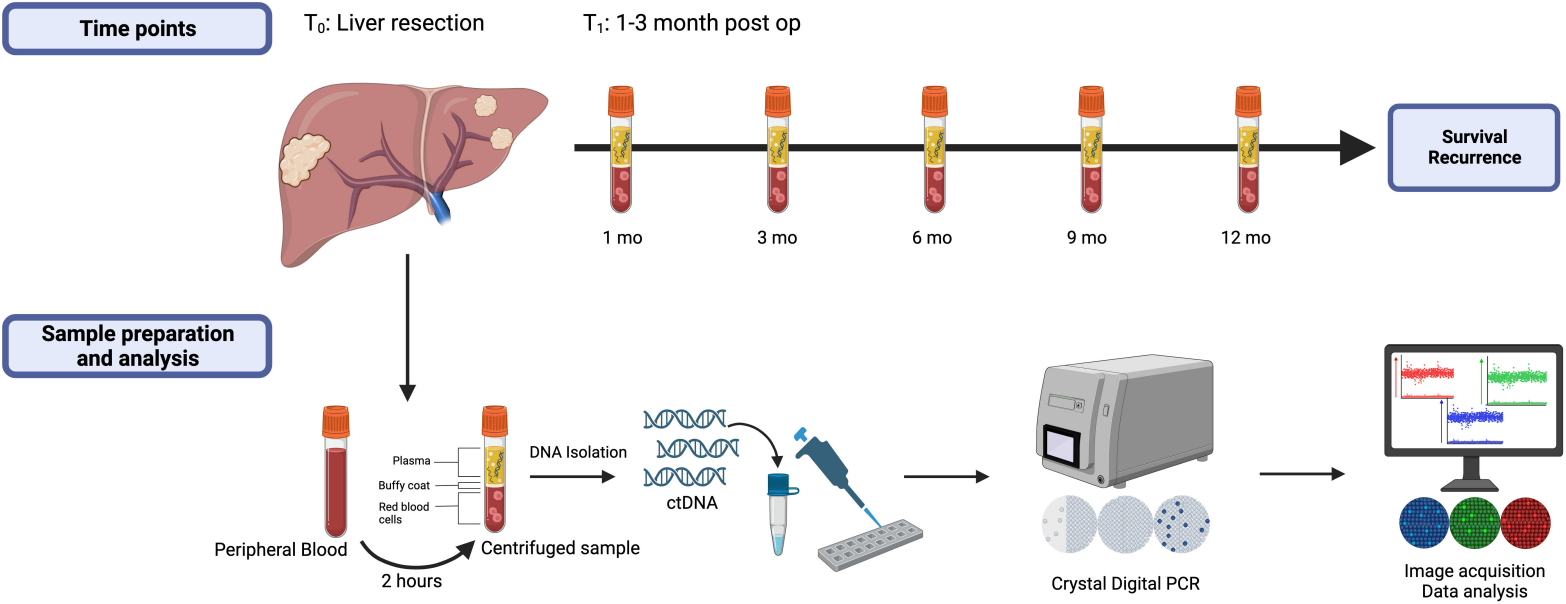
Study design highlighting the measured timepoints, blood sample preparation, and data analysis. All patients had samples drawn at time points T_0_ (baseline) and T_1_ (1-3 months post-liver resection) and were followed at least one year or until first recurrence.

Study design and details of blood collection are depicted in **Figure 2**. Blood samples were collected at initial referral or at time of consent for participation in the Liver Disease BioBank, in the operating room prior to liver resection (T_0_), 1-3 months (T_1_) following liver resection and every 3-6 months during post-operative surveillance. To maintain consistency across all samples and timepoints, the time of liver resection (T_0_) was designated as the initial time point used in this study and all patients had, at minimum, samples drawn at time of liver resection (T_0_) and at 1-3 months post liver resection (T_1_). Surveillance interval for the study lasted at least one year and up to 5 years. At each time point, 20 milliliters (mL) of blood were collected into two 10 mL EDTA tubes and were processed within 2 hours of collection at room temperature. The samples were centrifuged at 2000g for 15 minutes, plasma was separated, and the supernatant was aliquoted into 2 mL samples and stored at -80°C until DNA isolation.

### 2.4 ctDNA extraction and quantification

DNA was extracted and purified from 2 mL plasma samples using the QIAmp Circulating Nucleic Acid kit (Qiagen, Inc., Valencia, CA, USA) according to the manufacturer’s instructions. From each sample, 2 microliters (μL) were used to evaluate the concentration of cell-free DNA with the Qubit™ dsDNA High Sensitivity Quantification Assay kit (Thermo Fisher Scientific Inc., Waltham, MA, USA). Samples were stored at -20°C until further use.

### 2.5 Digital PCR assays for quantification of ctDNA

Detection and quantification of the KRAS and PIK3CA mutations using multiplex IDKRAS and IDPIK3CA kits (id Solutions, Grabels, France) was performed on the Naica^®^ System Crystal Digital PCR™ (Stilla Technologies, Villejuif, France) with the Sapphire Chip (Stilla Technologies, Villejuif, France). IDKRAS is a multiplex quantification system that allows for simultaneous amplification of KRAS mutation in codon G12, G13, and Q61, while IDPIK3CA allows for simultaneous amplification of the PIK3CA mutations at E542K, E545K, Q546K, H1047R and H1047L.

When multiple mutations were present, the mutation with the highest variant allele frequency as documented in the NGS report was identified. All samples were run in duplicate and 14 of 146 were run in triplicate due to low ctDNA concentration, as per Stilla protocol, to prevent false negatives. Likewise, samples with high concentration of ctDNA were diluted per Stilla protocol to prevent false positives.

The samples, plus a positive and negative control, were added to the Sapphire Chips and scanned using the 3-color Naica^®^ system (Prism3) with default exposure times for both KRAS and PIK3CA. Data was analyzed using the Stilla Crystal Miner Software (v2.4.0.3) and thresholds were set based on positive and negative controls for each mutation assay. Per the manufacture protocol, minimum thresholds of 5 detected mutant copies, calculated as a sum of the mutated droplets identified from both duplicated samples, or 7 detected mutant copies if the sample was run as triplicate, were established to determine if a sample was ctDNA positive for the specific mutation.

### 2.6 Statistical analysis

Demographic and clinicopathologic characteristics were summarized for all patients. Comparisons between groups according to ctDNA detection were performed using Fisher’s exact test or Chi-square test when appropriate. Logistic regression was performed to identify predictor variables or covariates effecting recurrence and ctDNA detectability and reported as odds ratio (OR). When there was 100% relationship between the predictor variable and outcome resulting in OR approaching infinity, Firth bias-reduction method was applied as described previously (18).

Cox proportional hazard models were used for univariate and multivariate analyses to identify factors associated with survival and reported as hazard ratio (HR). Survival analyses were estimated using Kaplan-Meier prediction and differences between groups were tested using log-rank test. Statistical analyses were run in GraphPad Prism (version 10.1.1 for macOS, GraphPad Software LLC, Boston, Massachusetts USA) and R Statistical Software (version 2023.12.0 for macOS, R Core Team 2022, Vienna, Austria).

## 3 Results

### 3.1 Clinicopathological characteristics and ctDNA analysis

During the study period, 213 patients with CRLM were identified from the Liver Disease BioBank that had undergone curative-intent liver resection. NGS data was available for 131 patients (61%), of which 85 (65%) had a KRAS and/or PIK3CA mutation. Of these, 29 patients met the inclusion criteria and had a KRAS and/or PIK3CA mutation which was detectable on the Naica^®^ System Crystal Digital PCR™ using the multiplexing IDKRAS and IDPIK3CA kits. A total of 115 blood samples were analyzed with an average of 3 postoperative samples per patient.

Baseline patient characteristics are summarized in **Table 1**. The median patient age was 61 years (range 44-80) and 58.6% were male. There was synchronous presentation of the liver metastases with the primary tumor in 25 (86%) patients. The median number of liver lesions present was 2 (range 1-8) and the median size of the largest lesion was 3.1 cm (range 0.8 – 17.9). All patients received perioperative chemotherapy with 24 of 29 (83%) receiving a median of 5 cycles (range 4-47) neoadjuvant treatment and 25 of 29 (86%) receiving a median of 6 cycles (range 1-15) adjuvant treatment. The histological subtype was adenocarcinoma in 23 patients (79.3%) with 2 of these showing a mucin-producing component, while 6 patients (20.7%) had mucinous adenocarcinoma. Histological differentiation was available for 24 patients, all of whom had moderately differentiated tumors. The viability of the liver tumor metastasis was a median of 30% (range 0-95%). Recurrence was diagnosed by cross-sectional imaging in 19 (65.5%) patients with a median time to recurrence of 9 months (range 1-48).

**Table 1:** Patient demographic and clinical characteristics.

Patients, n	29
Age (years)	61 (44-80)
Male: Female	17:12
CEA, at liver resection (ng/mL)	7 (1.4-163.4)
CEA, post liver resection (ng/mL)	2.45 (1.1-29.3)
Number of liver lesions	2 (1-8)
Diameter largest liver lesion (cm)	3.1 (0.8-17.9)
Location of colon primary Right Left Rectum	13 10 6
Synchronous presentation (%)	25 (86.2%)
Primary tumor N stage N0 N+ N/A	8 14 7
Chemotherapy Cycles, neoadjuvant Cycles, adjuvant	5 (0-47) 6 (0-15)
Histologic type Adenocarcinoma Mucinous Adenocarcinoma	23 (79.3%) 6 (20.7%)
Differentiation Moderate N/A	24 (82.8%) 5 (17.2%)
Tumor metastasis viability (%)	30 (0-95)
Recurrence (%)	19 (65.5)
First recurrence site (%) Liver Lung Liver + lung Other	5 (26.3) 8 (42.1) 2 (10.5) 4 (21)
Number of mutations 1 2 3	18 (62%) 8 (27.6%) 3 (10.3%)
Mutations present KRAS PIK3CA Other	28 (96.6%) 6 (20.1%) 9 (31%)

Data presented as median (range).

N/A, not available; BMI, body mass index; CEA, carcinoembryonic antigen.

NGS of the resected primary tumor or CRLM identified a mutation in every patient. There were 18 patients with a single KRAS mutation present and 11 patients with multiple mutations present. Of the 11 patients with multiple mutations, 2 had KRAS and ERBB2 mutations, 3 had KRAS and APC mutations, 2 had KRAS and PIK3CA mutations, 1 had BRAF and PIK3CA mutations, 2 had KRAS, PIK3CA and CTNNB1 mutations, and 1 had KRAS, PIK3CA, and MAP2K1 mutations.

### 3.2 Detection of preoperative ctDNA and association with DFS

The presence of ctDNA was detected in 16 of 29 (55%) preoperative samples. CEA levels were > 5 μg/L in 15 of 29 (52%) preoperative samples and 10 of these 29 (62.5%) also had detectable ctDNA. There was no association between a CEA level > 5 μg/L and the preoperative ctDNA status (OR 1.83, 95% CI 0.37-8.71; p=0.67). Neoadjuvant chemotherapy was administered in 12 of the 16 patients with detectable preoperative ctDNA and the remaining 4 patients had upfront liver resection. Of the 13 patients where ctDNA was not detected in the preoperative sample, 12 had received at least 4 cycles of neoadjuvant chemotherapy.

A series of Fisher’s exact tests and logistic regression analyses were performed to investigate the relationship between recurrence and several predictor variables including, preoperative ctDNA positivity, preoperative CEA level, number of liver lesions, size of largest liver lesion, synchronous presentation, neoadjuvant chemotherapy, and location of primary tumor. None of these variables were found to be statistically significant predictors of recurrence; however, the presence of ctDNA preoperatively had a trend towards significance (OR 1.62, 95% CI 0.85-1.91; p=0.056).

**Figure 3:**
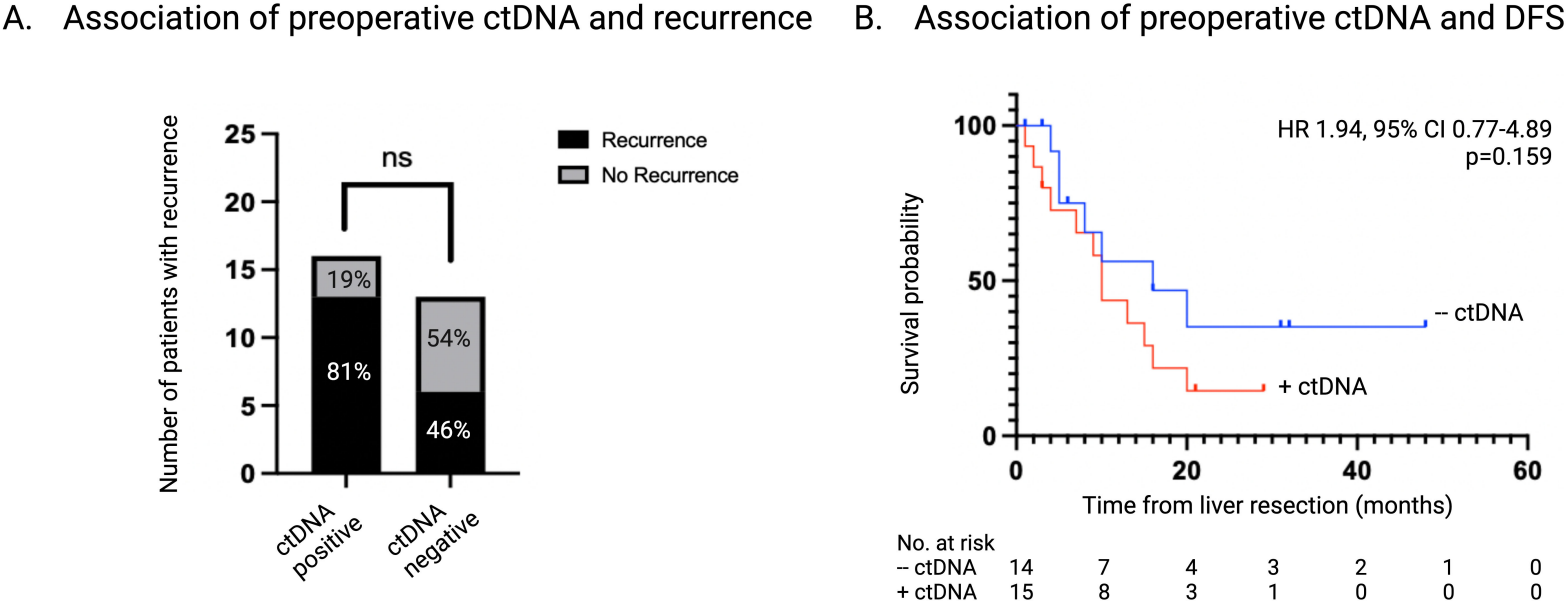
Association of ctDNA status at time of liver resection with recurrence. **(A)** Number and percentage of patients with recurrence stratified according to ctDNA status at time of liver resection. **(B)** Kaplan-Meier survival estimate of DFS stratified according to ctDNA status at time of liver resection. ctDNA, circulating tumor DNA; DFS, disease free survival.

Of the patients that had detectable ctDNA at the time of liver resection, 13 of 16 patients (81%) developed disease recurrence while 6 of 13 (46%) patients with undetectable ctDNA recurred (p=0.064) (**Figure 3A**). The median DFS for patients with detectable ctDNA preoperatively was 10 months vs 20 months for patients who had undetectable ctDNA preoperatively (HR 1.94, 95% CI 0.77-4.89; p=0.159) (**Figure 3B**).

### 3.3 Detection of postoperative ctDNA and association with DFS

**Figure 4:**
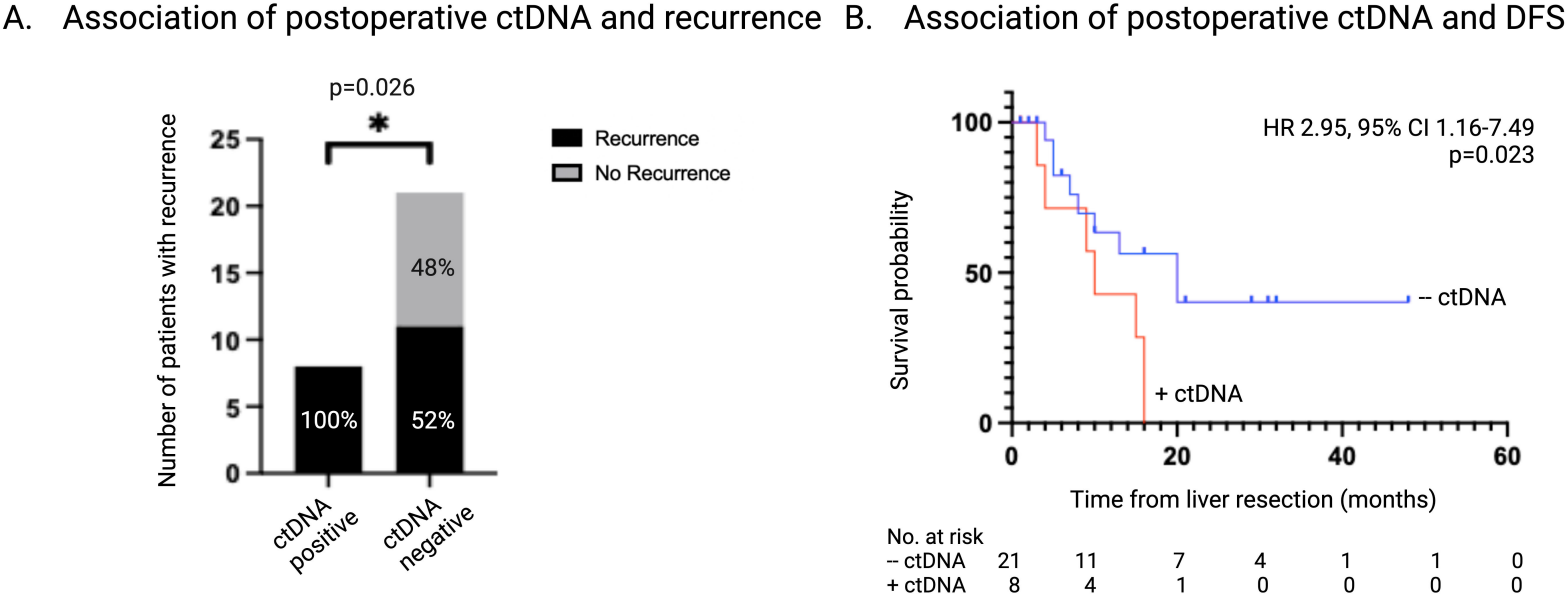
Association of ctDNA status 1-3 months postoperatively with recurrence. **(A)** Number and percentage of patients with recurrence stratified according to ctDNA status 1-3 months post-liver resection. **(B)** Kaplan-Meier survival estimate of DFS stratified according to ctDNA status 1-3 months post-liver resection. ctDNA, circulating tumor DNA; DFS, disease free survival.

The presence of ctDNA was detected in 8 of 29 (27.6%) postoperative samples, T_1_. All patients that had detectable ctDNA postoperatively also had detectable ctDNA at the time of liver resection. CEA levels were > 5ug/L in 4 of the 29 (13.7%) postoperative samples and 2 of these (50%) also had detectable ctDNA. There was no association between a CEA level > 5 μg/L and the postoperative ctDNA status (OR 3.8, 95% CI 0.47-27.4; p=0.25). Radiologic recurrence was diagnosed in all (8 of 8, 100%) of the patients who had detectable ctDNA postoperatively, compared to 11 of 21 patients (52%) with undetectable ctDNA postoperatively (p=0.026) (**Figure 4A**).

Further analysis was performed for the 11 patients who had undetectable ctDNA at T_1_ and went on to develop recurrence. Of these, 5 patients converted to having detectable ctDNA which coincided with the clinical diagnosis of recurrence. Sites of recurrence in these patients included one with liver only disease, two with lung only disease, and two with combined liver and lung disease. There were 6 patients with undetectable ctDNA at T_1_ who went on to develop recurrence without converting to having detectable ctDNA. Two patients had isolated pulmonary metastases and one of these patients also harbored an APC mutation. Three patients recurred in both the liver and lung and one of these patients also harbored an APC mutation. One patient had recurrent disease in the liver only which was diagnosed two months after the last available plasma sample.

Detectable ctDNA postoperatively was associated with a significantly shorter DFS of 9 months vs 13 months in patients who had no detectable ctDNA (HR 2.95, 95% CI 1.16-7.49; p=0.02) (**Figure 4B**). Logistic regression demonstrated a perfect separation between postoperative ctDNA presence and recurrence and therefore could not be effectively applied. Firth bias-reduction method was applied to the logistic regression model validating detectable ctDNA postoperatively is predictive of recurrence (OR 15.5, 95% CI 0.45-7.65; p=0.014). Univariate analysis found postoperative ctDNA status to be a significant prognostic factor in DFS (HR 3.23, 95% CI 1.24-8.42; p=0.017).

### 3.4 Detection of ctDNA and association with OS

**Figure 5:**
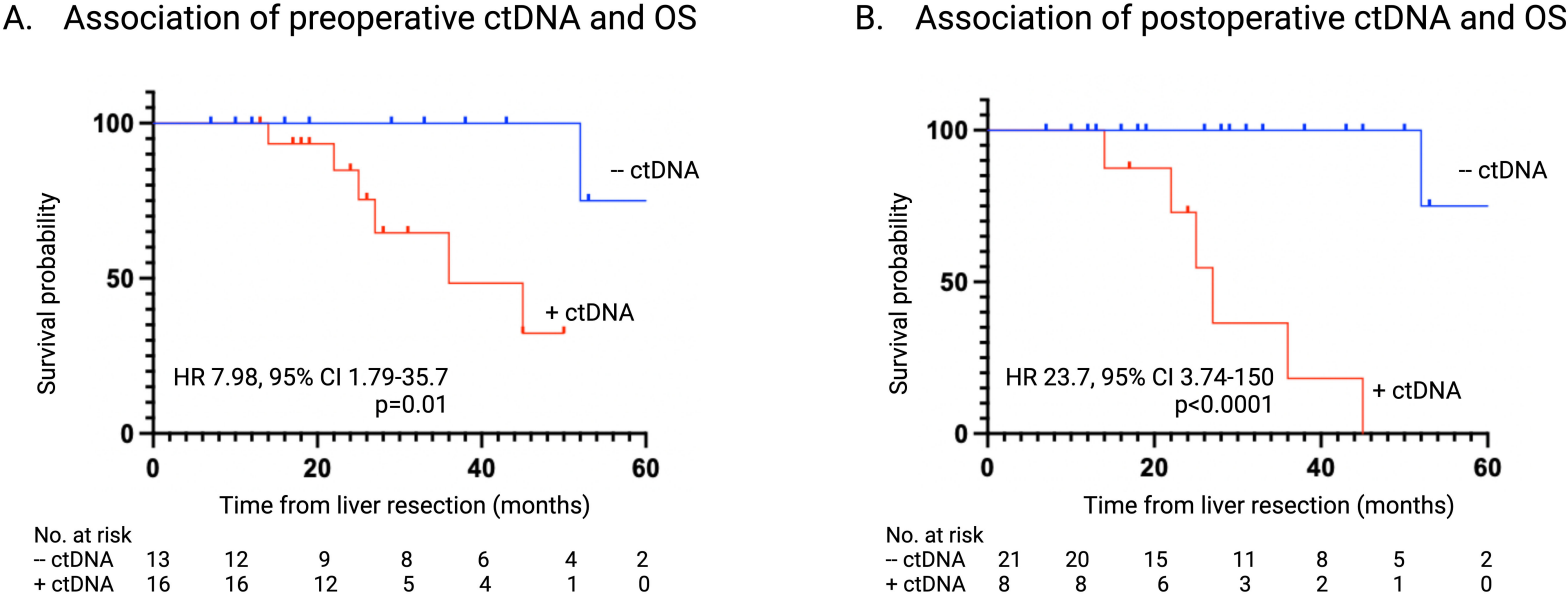
Association of ctDNA status on OS. **(A)** Kaplan-Meier survival estimate of OS stratified according to ctDNA status at time of liver resection. **(B)** Kaplan-Meier survival estimate of OS stratified according to ctDNA status 1-3 months post-liver resection. ctDNA, circulating tumor DNA; OS, overall survival.

The presence of detectable ctDNA regardless of timepoint (T_0_ vs T_1_) was associated with a significantly lower 5 year-OS compared to those with undetectable ctDNA (**Table 2**). The median survival of patients who were ctDNA negative at T_0_ and T_1_ was not reached, while the patients that had detectable ctDNA at T_0_ and T_1_ had a median survival of 36 months and 27 months, respectively (**Figure 5**).

**Table 2:** Impact of ctDNA status on median, OS, and DFS.

	T_0_ (baseline)		T_1_ (1-3 months post-LR)	
	– ctDNA	+ ctDNA	– ctDNA	+ ctDNA
**Median Survival (months)**	Not reached	36	Not reached	27
**5-year OS**	75%	32%	75%	0%
**HR (95% CI)**	Reference	7.98 (1.79-35.7)	Reference	23.7 (3.74-150)
**p value**		0.01		< 0.0001
**3-year DFS**	39%	14.5%	34.4%	0%
**HR (95% CI)**	Reference	1.94 (0.77-4.89)	Reference	2.95 (1.16-7.49)
**p value**		0.159		0.023

ctDNA, circulating tumor DNA; OS, overall survival; DFS, disease free survival; LR, liver resection.

## 4 Discussion

This study demonstrated that detectable ctDNA postoperatively was a strong predictor of recurrence as every patient who had detectable ctDNA at T_1_ developed recurrence. Because there was a perfect separation in the data, a Firth bias-reduction model was employed to mitigate the impact of detectable ctDNA postoperatively on recurrence. Despite this adjustment, the confidence interval for the odds ratio crosses one, indicating some uncertainty in the estimate’s precision, although the p value is 0.014, supporting a strong correlation in predicting recurrence. Although more patients who had detectable ctDNA preoperatively developed recurrence compared to those who had undetectable ctDNA (81% vs 46%, p=0.064; and an OR 1.62, p=0.056) this finding was not statistically significant. Additionally, this study established that ctDNA, whether detected pre or postoperatively, was associated with a lower median and overall survival compared to patients with undetectable ctDNA.

The findings in this study are consistent with previously published reports. A study by Bolhuis et al. (19) which included 23 patients with NRAS mutations evaluated the association between postoperative ctDNA and pathologic response and RFS after resection of CRLM. Of the 23 patients included, 17 (74%) developed recurrence during the median follow-up of 19.6 months. There were 11 patients (65%) with undetectable postoperative ctDNA who developed recurrence and 6 patients (100%) with detectable postoperative ctDNA who developed recurrence (19). Similarly, Marmorino et al. (14) conducted a study including patients with CRLM and used plasma samples to track mutations by dPCR using a panel. During the median follow up period of 77 months, 33 of 39 (85%) patients had detectable ctDNA, whereas 20 of 37 (54%) presented with undetectable ctDNA, all developed recurrence (p=0.008) (14). The significant relationship between detectable postoperative ctDNA and development of disease recurrence demonstrated across multiple studies, supports the use of ctDNA as a biomarker to detect MRD after resection for CRLM.

Despite these promising results, *how* ctDNA should be used clinically, particularly in the setting of CRLM, remains uncertain. Several studies have assessed how ctDNA changes with the administration of chemotherapy to help guide treatment decisions (17, 20–23). Previous studies have shown that when monitored in the neoadjuvant setting, ctDNA detection significantly decreases or even becomes undetectable by the fourth cycle of chemotherapy (20). While ctDNA clearance correlated with higher objective response rate on restaging imaging, it was not associated with improved RFS compared to patients with persistently detectable ctDNA preoperatively (20). Conversely, patients with undetectable ctDNA after surgery or those who cleared ctDNA during adjuvant treatment, had significantly improved RFS compared to those who had persistently detectable ctDNA postoperatively or during adjuvant treatment (5-year RFS 75.6% vs 0% and 66.7% vs 0%, respectively) (20). Few studies have evaluated ctDNA as a biomarker to guide postoperative management and have supported a role for selective use of adjuvant chemotherapy. In the GALAXY trial, part of the ongoing CIRCULATE-Japan study, analysis of preoperative and postoperative ctDNA status in patients with resectable stage II-IV colorectal cancer revealed higher risk of recurrence and worse DFS in patients with detectable postoperative ctDNA (22). Notably, patients with high-risk stage II or stage III disease and detectable postoperative ctDNA derived the greatest benefit from adjuvant chemotherapy (22). Additionally, treatment with adjuvant chemotherapy was associated with a higher incidence of ctDNA clearance compared to the patients who did not receive adjuvant chemotherapy. In the DYNAMIC trial, patients with stage II colorectal cancer were randomized to receive standard management or chemotherapy based on ctDNA presence and ctDNA-guided management was found to be noninferior (23). This approach led to a reduction in adjuvant chemotherapy use without compromising RFS (23). These data highlight the dynamic landscape of ctDNA and its potential for utilization in the clinical management of CRLM.

The detection of ctDNA after resection of CRLM has consistently been associated with increased rates of recurrence and decreased RFS (24). Interestingly, 30% - 60% of patients with undetectable ctDNA postoperatively will develop recurrence (14, 19, 20, 24). This may be due to tumor cells acquiring new mutations or altering existing ones as demonstrated by the mutation tracking concept used in breast cancer patients (25). A study applying this concept in patients with colorectal cancer tracked MRD by analyzing and tracking at least two variants in plasma (21). The authors found that by tracking two variants over serial plasma samples increased the accuracy by 35.3% (21). The patients included in the current study all harbored KRAS and/or PIK3CA mutations, but 4 of the 11 patients that developed recurrence and had undetectable ctDNA postoperatively also had additional mutations noted by NGS that were not being tracked during surveillance. Furthermore, it could be speculated that the patients with undetectable KRAS or PIK3CA ctDNA prior to liver resection, could have acquired a different mutation as these patients received at least 4 cycles of chemotherapy. The biology of the disease plays a crucial role in understanding treatment response. While ongoing treatment may lead to an absence of detectable ctDNA, tumor cells may still be present, albeit not actively shedding ctDNA. This necessitates a deeper understanding into the kinetics of ctDNA release and the emergence of treatment-resistant clones that may not be shedding ctDNA, due to perhaps being in a dormant state. The longer DFS observed in patients who initially had undetectable ctDNA at T_1_ but later showed detectable ctDNA shortly before developing recurrence, underscores the importance of periodic ctDNA monitoring. This pattern highlights ctDNA surveillance may be a pivotal tool for timely detection of disease progression and potentially lead to earlier intervention.

There are several limitations to this study inherent to its retrospective design. First, aside from T_0_ and T_1_, the number and timing of plasma samples was not consistent. The collection of blood was typically done at the follow up visits and due to a combination of missed appointments or delays in follow-up, not all the patients had the same time points after T_1_. Second, this study may be limited by the small sample size. Because 8 of 8 patients with detectable postoperative ctDNA developed recurrence, there was a potential for perfect separation in the logistic regression model necessitating the use of Firth bias-reduction model to mitigate its effects. Increasing the sample size may have alleviated this issue. Also, despite observing a higher recurrence rate in patients who were ctDNA-positive preoperatively compared to those who were ctDNA-negative, these results did not reach statistical significance, likely due to the limited sample size. Finally, many patients were referred after they had received at least four cycles of chemotherapy which prevented comprehensive analysis of the effects and dynamics of preoperative ctDNA status. The definition of detectable and undetectable ctDNA used in this study includes to only mutations in KRAS or PI3KCA and it is well established that CRLM present with clonal heterogeneity. Therefore, in the patient samples where KRAS or PI3KCA mutations are initially undetectable, the emergence of a different clonal variant and mutation profile could occur thereby eluding detection. Nonetheless, this study consists of a homogenous population and the findings are consistent with previously published data demonstrating the use of ctDNA as a biomarker to monitor MRD after surgery.

In conclusion, this study underscores the prognostic significance of postoperative ctDNA detection in predicting recurrence among patients undergoing resection for CRLM and contributes to the growing body of evidence supporting the role of ctDNA as a tool for personalized cancer management. Future studies are required to elucidate the optimal clinical utility using ctDNA-guided postoperative management strategies in the context of CRLM. This study also suggests that to effectively use ctDNA to predict recurrence, a multiplex panel is required. The next step of this study involves evaluating the same patient population with NGS at every timepoint to understand the dynamics of ctDNA release and clonal evolution in CRLM.

## Data Availability

The raw data supporting the conclusions of this article will be made available by the authors, without undue reservation.
